# Pharmacological influence on processes of adjuvant arthritis: Effect of the combination of an antioxidant active substance with methotrexate

**DOI:** 10.2478/v10102-012-0015-4

**Published:** 2012-06

**Authors:** Frantisek Drafi, Katarina Bauerova, Viera Kuncirova, Silvester Ponist, Danica Mihalova, Tatiana Fedorova, Juraj Harmatha, Radomir Nosal

**Affiliations:** 1 Institute of Experimental Pharmacology & Toxicology, Slovak Academy of Sciences, SK-84104 Bratislava, Slovakia; 2 Research Center of Neurology, RAMS, Moscow, Russia; 3 Institute of Organic Chemistry and Biochemistry, Academy of Sciences of the Czech Republic, v.v.i., Flemingovo namesti 2, 166 10, Praha, Czech Republic

**Keywords:** arthritis, oxidative stress, pinosylvin, carnosine, methotrexate, combination therapy

## Abstract

Oxygen metabolism has an important role in the pathogenesis of rheumatoid arthritis. A certain correlation was observed between oxidative stress, arthritis and the immune system. Reactive oxygen species produced in the course of cellular oxidative phosphorylation and by activated phagocytic cells during oxidative burst, exceed the physiological buffering capacity and result in oxidative stress. The excessive production of ROS can damage protein, lipids, nucleic acids, and matrix components. Patients with rheumatoid arthritis have an altered antioxidant defense capacity barrier. In the present study the effect of substances with antioxidative properties, i.e. pinosylvin and carnosine, was determined in monotherapy for the treatment of adjuvant arthritis (AA). Moreover carnosine was evaluated in combination therapy with methotrexate. Rats with AA were administered first pinosylvin (30 mg/kg body mass daily *per os*), second carnosine (150 mg/kg body mass daily *per os*) in monotherapy for a period of 28 days. Further, rats with AA were administered methotrexate (0.3 mg/kg body mass 2-times weekly *per os*), and a combination of methotrexate+carnosine, with the carnosine dose being the same as in the previous experiment. Parameters, i.e. changes in hind paw volume and arthritic score were determined in rats as indicators of destructive arthritis-associated clinical changes. Plasmatic levels of TBARS and lag time of Fe^2+^-induced lipid peroxidation (tau-FeLP) in plasma and brain were specified as markers of oxidation. Plasmatic level of CRP and activity of γ-glutamyltransferase (GGT) in spleen and joint were used as inflammation markers. In comparison to pinosylvin, administration of carnosine monotherapy led to a significant decrease in the majority of the parameters studied. In the combination treatment with methotrexate+carnosine most parameters monitored were improved more remarkably than by methotrexate alone. Carnosine can increase the disease-modifying effect of methotrexate treatment in rat AA.

## Introduction

Rheumatoid arthritis (RA) is characterized by persistent synovitis, systemic inflammation, and presence of autoantibodies. Genetic factors are implicated for 50% of the risk of developing RA. Smoking is the main environmental risk. In industrialized countries, RA affects 0.5–1.0% of adults, with 5–50 per 100,000 new cases annually. The disorder is most typical in women and elderly people. Uncontrolled active RA causes joint damage, disability, decreased quality of life, and cardiovascular and other comorbidities. Disease-modifying antirheumatic drugs (DMARDs), the key therapeutic agents, reduce synovitis and systemic inflammation and improve function of the musculoskeletal system. The leading DMARD is methotrexate, which can be combined with other DMARDs. Biological agents are used when arthritis is uncontrolled or toxic effects arise with DMARDs. Tumor necrosis factor alpha inhibitors were the first biological agents, followed by abatacept, rituximab, and tocilizumab. Infections and high costs restrict prescription of biological agents. RA has 19^th^ century roots. Its name was introduced in the 1850s. First classification criteria were developed only 50 years ago (Storey *et al.*, 1994; Ropes *et al.*, 1959; Arnett *et al.*, 1988). Several clinical studies as well as preclinical animal models of RA have documented an imbalance in the body redox homeostasis to a more pro-oxidative environment, suggesting that therapies that restore the balance have a beneficial effect (Kunsch *et al.*, 2005). Bauerova and Bezek (1999) and Jaswal *et al.* (2003) described oxidative stress as a primary factor involved in the RA pathogenetic changes. Our previous results with different antioxidants and immunomodulators showed a beneficial effect of these substances on the development of adjuvant arthritis (AA) (Bauerova *et al.*, 2005a, 2005b 2008a, 2008b 2009, 2011, Drabikova *et al.*, 2009; Drafi *et al.*, 2010; Jancinova *et al.*, 2009; Kogan *et al.*, 2005; Macickova *et al.*, 2010; Ponist *et al.*, 2010; Sotnikova *et al.*, 2009; Strosova *et al.*, 2008, 2009). The advantage of AA as an experimental arthritis model is its great similarity to RA, such as symmetrical joint involvement, persistent joint inflammation, synovial hyperplasia, and a good response to most therapies effective in RA (Bina & Wilder, 1999).

According to Babior (2000) reactive oxidants are essential tools for the pathogenesis of RA. The world of plants is an unlimited source of compounds with healing effects, including anti-inflammatory, antioxidative and immunomodulating properties. For this experimental overview, we chose to study the effect of pinosylvin (PIN). PIN [3′,5′-dihydroxystilbene] is a natural substance from the stilbenoid group, wide-spread in a variety of plants. PIN is chemically related to resveratrol, which is well known by its anti-oxidative activity. Structural analogues of resveratrol possess some of the beneficial effects of the parent drug and may provide even further benefits. PIN has been studied for its anticancer, antifungal and antioxidative properties (Roupe *et al.*, 2008). Further we chose the endogenous antioxidant carnosine (CARN) for monotherapy and as a suitable candidate for combination therapy with methotrexate (MTX). CARN is a dipeptide consisting of β–alanine and L-histidine. It was shown to be a specific constituent of excitable tissues of all vertebrates accumulating in amounts exceeding that of ATP (Boldyrev and Severin, 1990). The antioxidant capacity of this compound is well documented, as well as its pH buffering, osmoregulation, and metalchelating abilities (Boldyrev, 1990).

## Methods

### Animals, experimental design and treatments

Male Lewis rats, weighing 160–180 g, were obtained from the “Breeding Station Dobra Voda” (Slovakia). The rats had free access to standard pelleted diet and tap water *ad libitum*. The animal facilities comply with the European Convention for the Protection of Vertebrate Animals Used for Experimental and Other Purposes. The experimental protocol was approved by the Ethics Committee of the Institute of Experimental Pharmacology and Toxicology and by the State Veterinary and Food Administration of the Slovak Republic. Induction of arthritis in rats was performed by injecting a suspension of heat-killed *Mycobacterium butyricum* (MB) in incomplete Freund's adjuvant (Difco Laboratories, Detroit, MI, USA) intradermally into the tail base.

After 7-days quarantine, the animals were randomized as follows in 3 experiments:

1^st^ experiment included: 3 groups of eight – ten animals:

group 1 – healthy untreated controls (CO);

group 2 – untreated rats with adjuvant arthritis (AA);

group 3 – AA rats treated with carnosine (AA-CARN).

2^nd^ experiment included: 3 groups of eight – ten animals:

group 1 – healthy untreated controls (CO);

group 2 – untreated rats with adjuvant arthritis (AA);

group 3 – AA rats treated with pinosylvin (AA-PIN).

3^rd^ experiment included: 4 groups of eight – ten animals:

group 1 – healthy untreated controls (CO);

group 2 – untreated rats with adjuvant arthritis (AA);

group 3 – AA rats treated with methotrexate (AA-MTX);

group 4 – AA rats in combination therapy of carnosine and methotrexate (AA-CARN+MTX).

Pinosylvin (daily dose of 30 mg/kg b.w. *per os*) and carnosine (daily dose of 150 mg/kg b.w. *per os*) in monotherapy were administered to the arthritic animals from day 1 (day of arthritic insult) to the 28 (end of experiment). MTX in dose 0.3 mg/kg body mass was applied *per os* twice a week. The combination of carnosine and methotrexate (CARN – daily dose of 150 mg/kg b.w. *per os* + MTX in the dose of 0.3 mg/kg body mass twice a week *per os*) was administered to the animals during the whole experiment. PIN was synthesized and purified by Šmidrkal *et al.* (2010) and Harmatha *et al.* (2011). METHOTREXATE-TEVA^®^ 25 mg/ml (TEVA Pharmaceuticals Slovakia s.r.o.– SVK) was used. CARN was purchased from Hamary Chemicals Ltd., Japan. After the animals had been sacrificed under deep anesthesia, blood for plasma preparation and tissues for brain, spleen and hind paw joint homogenate preparation were taken on day 28. Heparinized plasma was stored at –70°C until biochemical and immunological analysis.

### Clinical parameters evaluated – hind paw volume change and arthrogram

Hind paw volume (HPV) was calculated as the percentage increase of the hind paw of each animal, compared to the HPV measured of the beginning of the experiment. HPV was recorded by means of an electronic water plethysmometer (UGO BASILE, Comerio-Varese, Italy). The arthritic score was measured as the total score of HPV (ml, max. points 8) + paw diameter of forelimb (mm, max. points 5) + diameter of scab, in the site of MB application, measured parallel to the spinal column (mm, max. points 5) for each animal.

### Tissue activity of cellular γ-glutamyltransferase in spleen and hind paw joint homogenates

The activity of cellular γ-glutamyltransferase (GGT) in hind paw joint and spleen tissue homogenates was measured by the method of Orlowski and Meister (1970) as modified by Ondrejickova *et al.* (1993). Samples were homogenized in a buffer (2.6 mM NaH_2_PO_4_, 50 mM Na_2_HPO_4_, 15 mM EDTA, 68 mM NaCl; pH 8.1) at 1:9 (w/v) by UltraTurax TP 18/10 (Janke & Kunkel, Germany) for 1 min at 0°C. Substrates (8.7 mM γ-glutamyl-p-nitroaniline, 44 mM methionine) were added to 65% isopropylalcohol to final concentrations of 2.5 mM and 12.6 mM, respectively. After incubation for 60 min at 37°C, the reaction was stopped with 2.3 ml cold methanol and the tubes were centrifuged for 20 min at 5,000 rpm. Absorbance of supernatant was measured in a Specord 40 (Jena, Germany) spectrophotometer in a 0.5 cm cuvette at 406 nm. Reaction mixtures in the absence of either substrate or acceptor were used as reference samples.

### Thiobarbituric acid reactive substances (TBARS) in plasma

The reaction with TBA occurs by attack of the monoenolic form of malondialdehyde (MDA) on the active methylene groups of TBA. Visible and ultraviolet spectrophotometry of the pigment confirms the primary maximum at 535 nm. TBARS were measured in heparinized blood plasma. The amount of 750 μL of 0.67% TBA (Merck), 750 μL of 20% trichloroacetic acid (Fluka), 350 μL of phosphate buffer (pH 7.4) were added to 150 μL of plasma, then mixed and incubated in a water bath at 90°C for 30 min. The reaction was stopped by dipping the test tubes into ice for 10 min. Samples were centrifuged at 3,000 rpm (centrifuge Eppendorf 5702 R, Germany). The supernatant was removed and absorbance measured at 535 nm (Specord 40, Jena, Germany) in a 0.5 cm cuvette.

### Lag time of Fe^2+^-induced lipid peroxidation (tau-FeLP) of plasma and brain

This measurement was analyzed using the chemiluminescence (CHL) signal derived from addition of ferrous ions to plasma and brain homogenates. After addition of 100 μl of 25 mM FeSO_4_ to the samples, the lag period between initial fast flash and the following slow rising CHL signal reflecting the rate of lipid oxidation was measured. The lag time (τ-tau) is referring to the stability of the sample to the Fe^2+^-induced oxidation (the longer the lag period the more stable the resistance of the biological material to oxidation) and is dependent on the intrinsic antioxidant capacity of plasma and brain. CHL signal was monitored using LKB 1251 Chemiluminometer (Sweden).

### C-reactive protein

For the determination of rat C-reactive protein (CRP) concentration in plasma the ELISA kit from Immunology Consultant Laboratories, Inc. (ICL) was used. The reaction of secondary biotin-conjugated anti-rat CRP antibody is evaluated by streptavidin-HRP. The tetramethyl-benzidine reaction with HRP bound to immune complex was measured at 450 nm (Microplate reader Labsystems Multiskan RC). The results were calculated using the standard calibration curve on internal standards.

### Statistical analysis

The data for all parameters are expressed as arithmetic mean ± S.E.M with eight – ten animals in each experimental group. For statistical analysis the unpaired Student‘s t-test was applied. The following symbols were used: *p<0.05 (significant), **p<0.01 (very significant), ***p<0.001 (extremely significant). The arthritis group (AA) was compared with healthy control animals (CO) (*). The treated arthritis groups in monotherapy (AA-PIN / AA-CARN) and the combination therapy (AA-CARN+MTX) were compared with untreated arthritis (AA) (+). The combination treatment (AA-CARN+MTX) was compared to the group treated with methotrexate (AA-MTX) (#).

## Results

Our results concern three experiments: 1^st^ monotherapy with pinosylvin (PIN), 2^nd^ monotherapy with carnosine (CARN), and 3^rd^ the combination therapy of carnosine and methotrexate (CARN+MTX).

**Figure 1:**
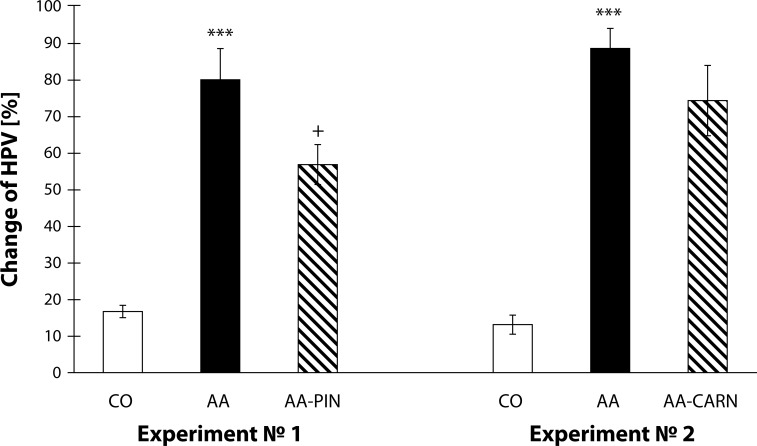
Change of HPV measured on day 28 in the model of adjuvant arthritis treated with pinosylvin and carnosine in monotherapy. CO – control group, AA – Adjuvant arthritis group, AA-PIN – Adjuvant arthritis group administered pinosylvin, AA-CARN – Adjuvant arthritis group administered carnosine. Hind paw volume (HPV) is expressed in percentages [%]. Results are mean ± S.E.M., n=8-10. The symbol (*) shows significant difference ***p<0.001 versus CO, +*p<*0.05 versus AA.

Figure 1 shows the results from the 1^st^ and 2^nd^ experiment on the clinical parameter change of HPV [%]. The change of HPV shows an increase in the arthritic groups compared to the control groups (AA vs. CO, ***p<0.001). Further the administration of pinosylvin decreased the change of HPV by 28.96% in comparison with the untreated arthritis group in a beneficially way (AA-PIN vs. AA, +*p<*0.05). In the second experiment, administration of carnosine decreased slightly the changed HPV by 16.09% in comparison with the untreated arthritis group (Figure 1).

**Figure 2:**
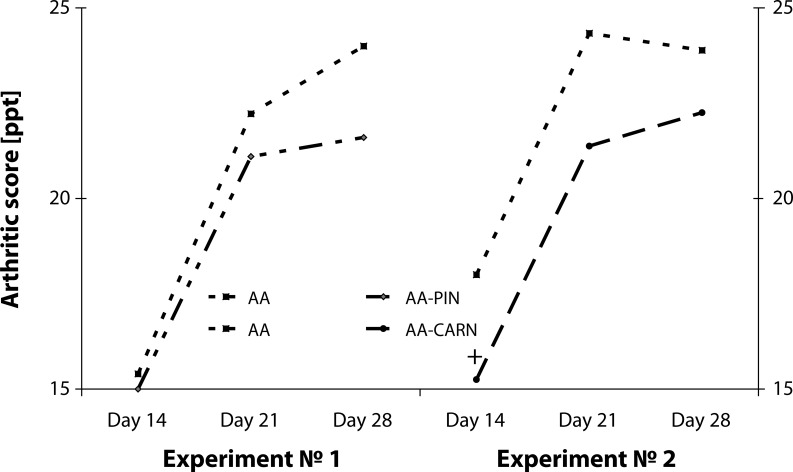
Arthritic score in the model of adjuvant arthritis treated with pinosylvin and carnosine in monotherapy. CO – control group, AA – Adjuvant arthritis group, AA-PIN – Adjuvant arthritis group administered with pinosylvin, AA-CARN – Adjuvant arthritis group administered carnosine. Arthrogram is expressed in points [pts]. Results are mean ± S.E.M., n=8-10. The symbol (+) shows significant difference +*p<*0.05 vs AA.

The points of arthritic score in Figure 2 show a trend to decrease the score for the treated groups in both experiments administered PIN or CARN in comparison to untreated arthritic animals. With CARN administration, the effect was clearly significant only on day 14 (AA-CARN vs. AA, +*p<*0.05) (Figure 2).

**Figure 3:**
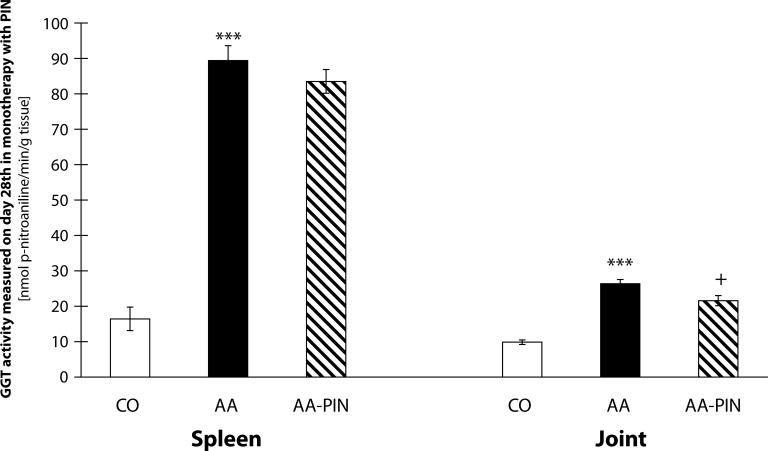
GGT activity in spleen and joint on day 28 in the model of adjuvant arthritis treated with pinosylvin in monotherapy. CO – control group, AA – Adjuvant arthritis group, AA-PIN – Adjuvant arthritis group administered with pinosylvin. The activity of cellular γ-glutamyltransferase (GGT) in hind paw joint and spleen tissue homogenates is expressed in [nmoln p-nitroaniline/min/g tissue]. Results are mean ± S.E.M., n=8-10. The symbol (*) shows significant difference ***p<0.001 vs CO, +*p<*0.05 vs AA.

The results in Figure 3 show the activity of cellular γ-glutamyltransferase (GGT) in hind paw joint and spleen tissue homogenates on PIN administration. The value of the activity of GGT in the spleen in the 1^st^ experiment shows an increase in the arthritis group compared to the control group (AA vs. CO, ***p<0.001). Administration of PIN decreased the activity of GGT in spleen by 6.58% compared with the untreated arthritis group. Further, the value of the activity of GGT increased significantly in the joint in the 1^st^ experiment in the arthritis group compared to the control group (AA vs. CO, ***p<0.001). Administration of pinosylvin decreased the activity of GGT in the joint by 18.14% in comparison with the untreated arthritis group (AA-PIN vs. AA, +*p<*0.05) (Figure 3).

**Figure 4:**
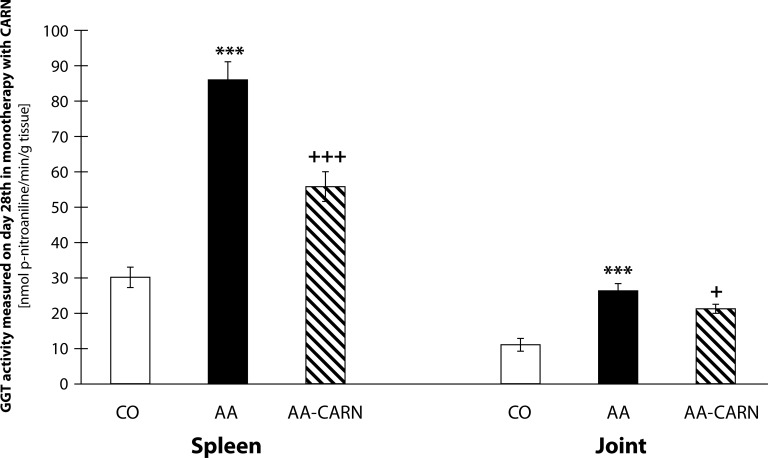
GGT activity in spleen and joint on day 28 in the model of adjuvant arthritis treated with carnosine in monotherapy. CO – control group, AA – Adjuvant arthritis group, AA-CARN – Adjuvant arthritis group administered carnosine. The activity of cellular γ-glutamyltransferase (GGT) in hind paw joint and spleen tissue homogenates is expressed in [nmoln p-nitroaniline / min / g tissue]. Results are mean ± S.E.M., n=8-10. The symbol (*) shows significant difference ***p<0.001 vs CO, +*p<*0.05 vs AA, +++*p<*0.001 vs AA.

In the 2^nd^ experiment (Figure 4), the activity of GGT in the spleen shows significant increase in the arthritis group compared to the control group (AA vs. CO, ***p<0.001). Administration of CARN decreased significantly the activity of GGT in the spleen by 35.17% compared with the untreated arthritis group (AA-CARN vs. AA, +++*p<*0.001). The activity of GGT in the joint showed also significant increase in the arthritis group compared to the control group (AA vs. CO, ***p<0.001). Administration of carnosine decreased significantly the activity of GGT in the joint by 19.30% compared with the untreated arthritis group (AA-CARN vs. AA +*p<*0.05). The GGT activity assessed in the joint homogenate achieved results which showed the same trend in both experiments (Figure 4).

**Figure 5:**
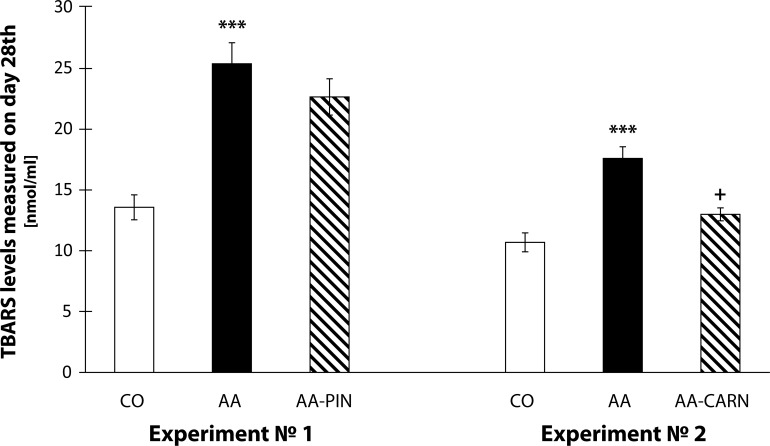
TBARS measured on day 28 in the model of adjuvant arthritis treated with pinosylvin and carnosine in monotherapy. CO – control group, AA – Adjuvant arthritis group, AA-PIN – Adjuvant arthritis group administered with pinosylvin, AA-CARN – Adjuvant arthritis group administered with carnosine. Levels of TBARS in plasma are expressed in [nmoln/ml]. Results are mean ± S.E.M., n=8–10. The symbol (*) shows significant difference ***p<0.001 vs CO, +*p<*0.05 vs AA.

The results in Figure 5 show the levels of plasmatic TBARS measured on day 28 [nmol/ml]. The levels of plasmatic TBARS increased significantly in the 1^st^ experiment in the arthritis group compared to the control group (AA vs. CO, ***p<0.001). Administration of pinosylvin decreased the level of TBARS in plasma by 10.76% compared with the untreated arthritis group (AA-PIN vs. AA). In the 2^nd^ experiment, the increased levels of TBARS measured on day 28 showed also significance in the arthritis group compared to the control group (AA vs. CO, ***p<0.001). Administration of carnosine decreased significantly the level of TBARS in plasma by 26.11% in comparison with the untreated arthritis group (AA-CARN vs. AA, +*p<*0.05). The results show that carnosine improved more efficiently the TBARS levels in plasma than PIN (Figure 5).

**Figure 6:**
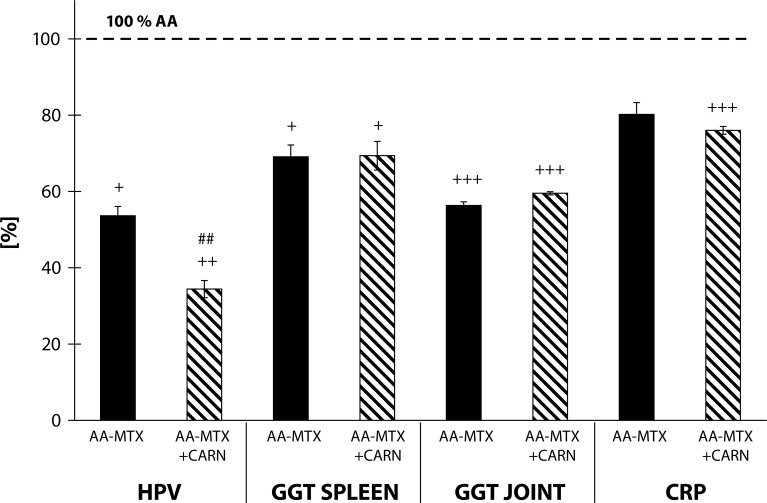
Effect of methotrexate and the combination of carnosine + methotrexate on adjuvant arthritis on the parameters of hind paw volume (HPV), activity of cellular γ-glutamyltransferase (GGT) in hind paw joint and spleen tissue homogenates and C-reactive protein (CRP) in plasma measured on day 28, expressed in percentage compared to the arthritis group considered as 100%. AA – Adjuvant arthritis group (100%), AA- MTX – Adjuvant arthritis group administered methotrexate and AA-CARN+MTX – Adjuvant arthritis group administered with carnosine+methotrexate. Results are mean ± S.E.M., n=8–10. The symbol (+) shows significant difference +*p<*0.05 vs AA, ++*p<*0.01 vs AA, +++*p<*0.001 vs AA, ##*p<*0.01 vs AA-MTX.

The results in Figure 6 show the values from the 3^rd^ experiment on selected clinical and inflammation parameters, i.e. change of HPV, GGT activity (spleen and joint homogenates) and levels of C-reactive protein (CRP) in plasma. The HPV decreased in the arthritis group treated with MTX by 46.38% in comparison to AA (AA-MTX vs. AA, +*p<*0.05). Administration of carnosine+methotrexate decreased HPV by 65.59% in comparison with the untreated arthritis group (AA-CARN+MTX vs. AA, ++*p<*0.01). The activity of GGT decreased by 30.93% in the spleen in the arthritis group treated with MTX in comparison to AA (AA-MTX vs. AA, +*p<*0.05). Administration of carnosine+methotrexate decreased the activity of GGT by 30.62% compared to the untreated arthritis group (AA-CARN+MTX vs. AA, +*p<*0.01). In the arthritis group treated with MTX the activity of GGT in the joint decreased by 43.38% significantly in comparison to AA (AA-MTX vs. AA, +++*p<*0.001). Administration of carnosine+methotrexate significantly decreased the activity of GGT by 40.47% in comparison to the untreated arthritis group (AA-CARN+MTX vs. AA, +++*p<*0.001). The level of CRP decreased by 19.78% in plasma measured on day 28 in the arthritis group treated with MTX in comparison to AA. Administration of carnosine+methotrexate decreased the level of CRP in plasma by 23.98% in comparison to the untreated arthritis group (AA-CARN+MTX vs. AA, +++*p<*0.001). Figure 6 shows a statistically significant difference between MTX monotherapy used as reference treatment and its combination with CARN only for the clinical parameter – change of HPV.

**Figure 7:**
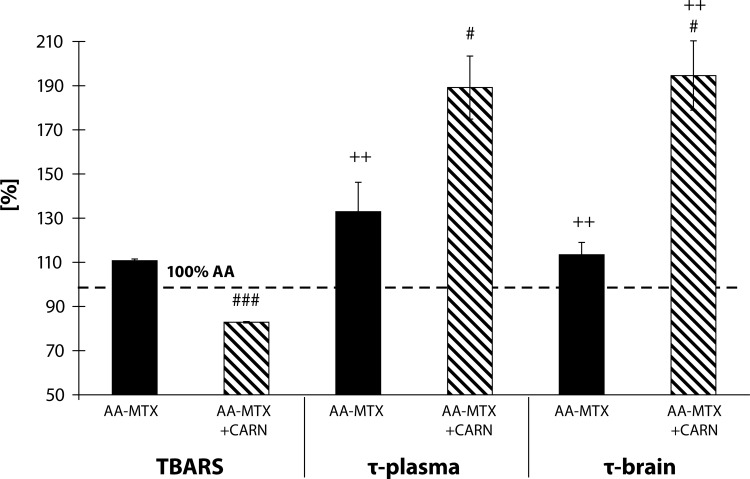
Effect of methotrexate and the combination of carnosine + methotrexate on adjuvant arthritis on the parameters of TBARS, tau-FeLP in plasma and brain on day 28, expressed in percentages in comparison to the arthritis group considered as 100%. AA – Adjuvant arthritis group (100%), AA- MTX – Adjuvant arthritis group administered methotrexate and AA-CARN+MTX – Adjuvant arthritis group administered carnosine+methotrexate. Results are mean ± S.E.M., n=8–10. The symbol (+) shows significant difference ++*p<*0.01 vs AA, #*p<*0.05 vs AA-MTX, ###*p<*0.001 vs AA-MTX.

The results in Figure 7 show the values from the 3^rd^ experiment on the parameters: plasmatic levels of TBARS, lag time of Fe^2+^-induced lipid peroxidation (tau-FeLP) in plasma and brain expressed in percentages in comparison to the arthritis group considered as 100%. The levels of TBARS in the arthritis group treated with MTX increased by 10.76% in comparison to AA. Administration of carnosine+methotrexate decreased the level of TBARS by 17.16% in comparison with the arthritis group. In plasma in the arthritis group treated with MTX, the tau-FeLP was increased by 32.96% in comparison to AA (AA-MTX vs. AA, ++*p<*0.01). Administration of carnosine+methotrexate increased significantly the tau-FeLP plasma in comparison to the untreated arthritis group by 89.14% (AA-CARN+MTX vs. AA, ++*p<*0.01). In the arthritis group treated with MTX, the lag time of Fe^2+^-induced lipid peroxidation in the brain was increased significantly in comparison to AA by 13.43% (AA-MTX vs. AA,++*p<*0.01). Administration of carnosine+methotrexate increased significantly the lag time of Fe^2+^-induced lipid peroxidation in the brain in comparison to the untreated arthritis group by 94.53% (AA-CARN+MTX vs. AA, ++*p<*0.01). For all oxidative stress parameters we found a statistically significant difference between MTX monotherapy used as reference treatment and its combination with CARN.

## Discussion

In the presented experiments we showed the effect of two antioxidative substances tested (pinosylvin and carnosine) and the combination of the latter one with methotrexate in the model of adjuvant arthritis. Pinosylvin in monotherapy decreased signigficantly the basic clinical parameter HPV in comparison to untreated animals. Our previous experiments (Bauerova *et al.*, 2007, 2008a, 2008b 2009, Rovensky *et al.*, 2008, Rovensky *et al.*, 2009a) reported that clinical parameters, such as HPV and body weight, became significantly worse due to arthritis and were efficiently decreased by administration of substances with antioxidative properties. The administration with carnosine did not exert significant effect on HPV but showed a potency to decrease the changes in HPV (Figure 1). On the other hand, the combination of carnosine with methotrexate decreased the HPV more effectively than did treatment with methotrexate alone (Figure 6). This result is in compliance with other studies on combination of naturally occurring substances and methotrexate, where the combination proved more effective than monotherapy (Bauerova *et al.*, 2009, 2010; Rovensky *et al.*, 2002, 2009a, 2009b). The substances pinosylvin and carnosine, failed to decrease significantly the arthritic score in comparison to untreated arthritic animals (Figure 2). This is due to the fact that the arthritic score consists of five different inputs (two forelimbs, two hind limbs and scab) and these are influenced variably.

The activity of GGT is considered a reliable biochemical marker of oxidative stress as it is sensitive to immunomodulating effects of yeast polysaccharides (Bauerova *et al.*, 2006). Ishizuka *et al.* (2007) found that neutralizing antibodies against GGT had a therapeutic effect on joint destruction in collagen-induced arthritis in mice. Increased GGT activity was observed in serum and also in the synovial fluid of affected joints in patients with RA (Rambabu *et al.*, 1990) and also in joints of AA-animals (Ponist *et al.*, 2010). Previously we found that the activity of GGT was approximately 3–6 times higher in AA animals than in healthy controls in the spleen and 1.4–2.3 higher in the joint (Bauerova *et al.*, 2006; 2008a; 2009; Sotnikova *et al.*, 2009). Elevated expression and activity of GGT in joint tissue is a good marker for synovial inflammation and bone resorption, thus molecules able to reduce the activity and/or expression of GGT could be important for RA therapy. In our study, we observed increased activity of GGT in joint tissues and also in the spleen in animals with induced AA. Administration of pinosylvin slightly decreased the activity of GGT in the spleen (Figure 3). On the other hand, administration with carnosine remarkably decreased the activity of GGT in this important organ of the immune system (Figure 4). After pinosylvin or carnosine administration to arthritic rats, the activity of GGT in joint tissue decreased significantly in the same manner for both compounds tested (Figures 3, 4). Administration of MTX led to significantly decreased values of GGT activity in the spleen and joint (Figure 6). This beneficial effect of MTX was reported also in our previous experiments (Feketeova *et al.*, 2011; Bauerova *et al.*, 2010; Ponist *et al.*, 2010; Jurcovicova *et al.*, 2009) with a similar experimental design and setting. The combination of carnosine with methotrexate failed to yield a better effect on the improvement of GGT activity in the spleen and joint than did methotrexate treatment alone (Figure 6).

A frequently used marker of lipid peroxidation is MDA assessed as an adduct with TBA. Clinical studies have shown increased plasmatic levels of MDA in patients with RA (Baskol *et al.*, 2005; 2006; Sarban *et al.*, 2005). Also in animal models of AA, an increase in oxidative stress, represented by evaluated plasmatic levels of TBARS, was detected (Tastekin *et al.*, 2007; He *et al.*, 2006; Bauerova *et al.*, 2008b; 2009; Strosova *et al.*, 2008, 2009; Sotnikova *et al.*, 2009). In our previous work using the model of AA we found evidence of systemic oxidative stress, specifically: increased protein carbonyls (Bauerova *et al.*, 2005a), increased lipid peroxidation (Bauerova *et al.*, 2005b), and a significant reduction of total antioxidant status (Mihalova *et al.*, 2007) in plasma. Measurements of plasma TBARS in the present study confirmed that AA induced a systemic oxidative damage. Administration of carnosine was more effective than pinosylvin in reducing this oxidative damage monitored as TBARS levels in plasma (Figure 5). Moreover the combination of carnosine+methotrexate was more effective than methotrexate alone (Figure 7).

The levels of C-reactive protein (CRP), a protein found in plasma, rise in response to inflammation (Thompson *et al.*, 1999). Methotrexate decreased significantly the level of CRP in plasma of arthritic animals, as described by Bauerova *et al.* (2011). The level of CRP in AA rats was even more decreased by the combination therapy of carnosine+methotrexate (Figure 6). Furthermore, the group with combination therapy of carnosine and methotrexate increased the lag time of Fe^2+^-induced lipid peroxidation (tau-FeLP) in plasma and in brain samples, which refers to its ability to restore the systemic antioxidant capacity in plasma and locally in brain tissue (Figure 7). Carnosine exhibited a good protective activity against Fe^2+^-induced lipid peroxidation of human plasma lipoproteins *in vitro* and in the brain of experimental animals (Dobrota *et al.*, 2005; Fedorova *et al.*, 1999). In the present experiment, we report for the first time the protective effect of carnosine in combination therapy on tau-FeLP in Lewis rats with AA (Figure 7).

The results of our investigation confirmed the previously reported effect of methotrexate treatment in rats with AA (Welles *et al.*, 1985; Connolly *et al.*, 1988). Methotrexate at a dose of 0.6 mg/kg body mass per week (Bauerova *et al.*, 2010) suppressed but did not prevent arthritis development. The therapeutic effect of MTX was confirmed also by a significant reduction in the edema of the affected joints. Such effect could be ascribed also to the oxidative stress reducing properties of MTX (Koner *et al.*, 1997), as already reported in patients with RA (Herman *et al.*, 2008; Kageyama *et al.*, 2007). The anti-inflammatory effect of MTX is accompanied with suppression of oxidative burst of stimulated blood phagocytes in animals treated with MTX and was proved in our previous experiments (Bauerova *et al.*, 2010; Nosal *et al.*, 2007). Finally, the reduction of hind paw volume and CRP were more pronounced in rats treated with the combination of carnosine+methotrexate than in those treated with methotrexate alone. Moreover, comparable results were observed for TBARS levels in plasma. Thus we can conclude that oxidative stress plays an important role in AA and could be controlled by suitable combination therapy of MTX and an antioxidant substance, as demonstrated for carnosine.
